# Successful treatment in a case of *Propionibacterium acnes*-associated sarcoidosis with clarithromycin administration: a case report

**DOI:** 10.1186/1752-1947-8-15

**Published:** 2014-01-15

**Authors:** Nobuo Takemori, Masaya Nakamura, Masaru Kojima, Yoshinobu Eishi

**Affiliations:** 1 Division of Hematology, Department of Internal Medicine, Imai Hospital, Tanaka-cho 100, Ashikaga, Tochigi 326-0822, Japan; 2 Department of Surgery, Imai Hospital, Tanaka-cho 100, Ashikaga, Tochigi 326-0822, Japan; 3 Department of Anatomic and Diagnostic Pathology, Dokkyo Medical University School of Medicine, Mibu-machi, Shimotsuga-gun, Tochigi 321-0293, Japan; 4 Department of Human Pathology, Tokyo Medical and Dental University, 1-5-45, Yushima, Bunkyo-ku, Tokyo 113-8510, Japan

**Keywords:** Apoptosis, Clarithromycin (CAM), *Propionibacterium acnes* (*P. acnes*), Sarcoid granuloma, Sarcoidosis, Treatment

## Abstract

**Introduction:**

Sarcoidosis is recognized as a multiorgan disorder characterized by the presence of non-caseating granulomas in the involved tissues. It has been suggested that sarcoidosis might be due to the exposure to infectious or non-infectious agents in genetically susceptible individuals. In particular, *Propionibacterium acnes* and *Mycobacterium tuberculosis* have been considered causative microorganisms. We report a case of *P. acnes*-associated sarcoidosis in which a drastic improvement was achieved with clarithromycin administration. A possible mechanism of clarithromycin action is discussed.

**Case presentation:**

A 78-year-old Japanese-Mongoloid woman with *P. acnes*-associated sarcoidosis presented with a persisting fever, joint pains and generalized lymph node swelling. The diagnosis of sarcoidosis was confirmed by pathological and immunohistochemical studies of a biopsied lymph node. In this case, an oral administration of clarithromycin was applied. Soon after the initiation of this treatment her symptoms as well as lymph node swelling disappeared. The clarithromycin treatment was discontinued 3.5 months after its initiation. She is currently in good condition. The pathological analysis of her lymph node, which was obtained during the clarithromycin treatment, suggested an apoptosis-inducing effect of clarithromycin on the sarcoid granulomas.

**Conclusions:**

Clarithromycin was found to be effective for treating sarcoidosis and seems to have important pharmacological effects such as immunosuppression, immunomodulation and induction of apoptosis in addition to its antimicrobial role. In this case, apoptosis in the sarcoid granulomas induced by clarithromycin administration might have resulted in satisfactory improvement.

## Introduction

Sarcoidosis is recognized as a multiorgan disorder characterized by the presence of non-caseating granulomas in the involved tissues [1]. The disease involves predominantly the skin, eyes, lungs and lymph nodes, especially the hilar lymph nodes. Many studies of sarcoidosis have suggested that it might be due to the exposure to infectious or non-infectious agents in genetically susceptible individzuals [1,2]. In particular, *Propionibacterium acnes* (*P. acnes*) and *Mycobacterium tuberculosis* have been studied extensively as causative microorganisms. Latent infection of *P. acnes* has been emphasized as a cause of sarcoidosis [3], since *P. acnes* is the only microorganism that has been isolated from sarcoid lesions by bacterial culture [4].

Clarithromycin (CAM), a member of the macrolide family, is a widely used antimicrobial drug. It is also known to have other important pharmacological effects such as immunosuppression or immunomodulation [5,6]. We report here a rare case of sarcoidosis in which a drastic improvement was achieved with CAM administration. A possible mechanism of CAM action including its antimicrobial, immunosuppressive, immunomodulatory and apoptosis-inducing effects in the treatment of sarcoidosis is discussed.

## Case presentation

**Figure 1:**
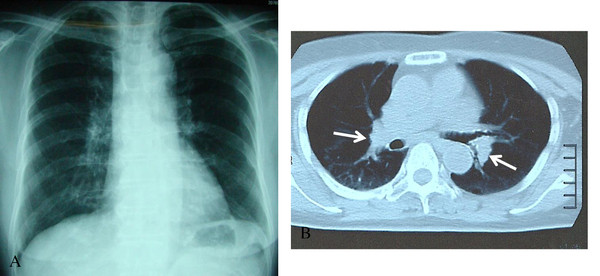
**Chest X-ray and computed tomography scan images on admission. (A)** Chest X-ray showing minimal bilateral hilar lymphadenopathy with clear lung fields. **(B)** Chest computed tomography scan clearly showing mild bilateral hilar lymphadenopathy (arrows) without pulmonary infiltrates.

A 78-year-old Japanese-Mongoloid woman (body weight: 48kg) with a 3-year history of hypertension and hyperlipidemia visited our hospital because of a persisting fever and generalized joint pains which had developed 2 weeks before initial presentation. She had been treated as having a common cold at a local clinic for 2 weeks previously, but her symptoms had not improved. After visiting our hospital, a drip infusion therapy of cefpirome with oral administration of loxoprofen was initiated on an out-patient basis. However, this treatment was not effective for the fever but caused toxic eruptions on the back of both her hands. Thus, this treatment was suspended 3 days later. She was admitted 4 days after initial presentation. Neither respiratory nor ocular symptoms were present. She had past history of panhysterectomy due to uterine cancer at the age of 38 and a fracture of her left wrist joint at the age of 68. She had no environmental or occupational history of beryllium or other metal exposure. A physical examination on admission showed bilateral inguinal and axillary lymph node swelling and erythematous eruptions on the back of both her hands. A chest X-ray showed minimal bilateral hilar lymphadenopathy (BHL); however, a chest computed tomography (CT) scan clearly revealed mild BHL without pulmonary infiltrates (Figure 1). Since respiratory function tests were normal, bronchoscopy was not performed. Electrocardiogram and ophthalmologic evaluations were normal. A complete blood cell count showed slight anemia (red blood cell count, 3.70×10^12^/L; hemoglobin, 10.8g/dL), slight leukocytosis (white blood cell count, 11.9×10^9^/L with 74% neutrophils, 13% lymphocytes, 11% monocytes, 1% eosinophils, and 1% basophils) and normal platelet count (360×10^9^/L). Elevated levels of erythrocyte sedimentation rate (110mm/hour), C-reactive protein (CRP; 13.73mg/dL; normal range 0 to 0.26mg/dL), soluble-interleukin (IL)-2 receptors (s-IL2R; 1300IU/mL; normal range 124 to 466IU/mL), antinuclear antibodies (×640; normal range <×40) and ferritin (722ng/mL; normal range 39.4 to 340ng/mL), and reduced levels of serum iron (34μg/dL; normal range 54 to 181μg/dL) and albumin (2.5g/dL; normal range 3.9 to 4.9g/dL) were observed. Serum electrolytes and renal function indices were normal. Rheumatoid arthritis particle agglutination, anti-double-stranded deoxyribonucleic acid (DNA), anti-Sm, anti-thyroglobulin, anti-microsome, anti-Ro and anti-La antibody titers were within normal limits. No increments of serum angiotensin-converting enzyme and lysozyme were seen. The results of an anti-acid fast bacterium antibody and a tuberculin skin test were negative (0×0mm). Serologic tests for syphilis, hepatitis B virus, hepatitis C virus and human immunodeficiency virus were negative. Serum Epstein–Barr virus (EBV) and *Chlamydia pneumoniae* titers showed prior infection patterns. The results of serologic studies for *Cytomegalovirus*, *Brucella*, *Legionella*, *Coxiella burnetii, Mycoplasma* and *Toxoplasma* were negative*.* Her urine showed nothing remarkable.

**Figure 2:**
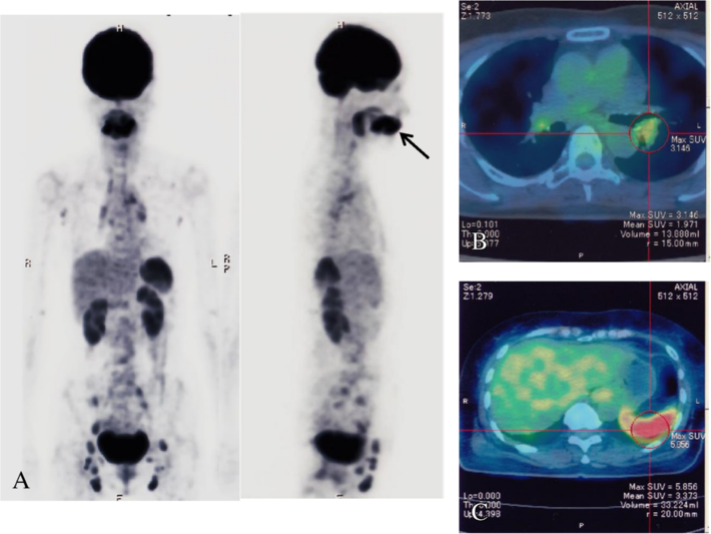
**Positron emission tomography-computed tomography images on admission. (A)** Coronal and sagittal positron emission tomography images on admission showing ^18^F-fluorodeoxyglucose uptake in peripharyngeal, axillary, mediastinal, hilar, iliac and inguinal lymph nodes and spleen. An arrow indicates ^18^F-fluorodeoxyglucose uptake at submandibular dental roots, suggesting submandibular periodontitis. **(B)** Fused positron emission tomography-computed tomography image showing left hilar lymph node swelling with moderate ^18^F-fluorodeoxyglucose uptake. **(C)** Fused positron emission tomography-computed tomography image showing remarkable ^18^F-fluorodeoxyglucose uptake in the spleen.

A positron emission tomography-CT (PET-CT) scan, which was carried out 4 days after admission, showed ^18^F-fluorodeoxyglucose (FDG) uptakes in her peripharyngeal, axillary, mediastinal, hilar, iliac and inguinal lymph nodes with splenic involvement. In addition, remarkable FDG uptake at her submandibular dental roots wearing ceramic crowns was observed, suggesting chronic periodontitis (Figure 2).

**Figure 3:**
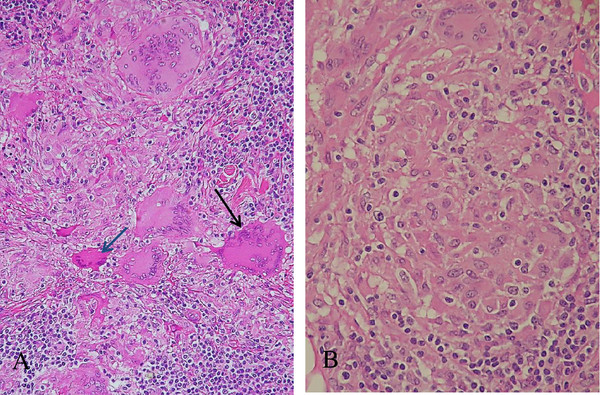
**Histopathology of the lymph node. (A)** Langhans giant cells with various degrees of eosin stainability are seen. One giant cell (black arrow) shows moderately eosinophilic condensed cytoplasm, and another one (blue arrow) has a remarkably eosinophilic condensed cytoplasm with multiple condensed nuclei. Hematoxylin and eosin stain (under×20 magnification objective). **(B)** The sarcoid granuloma consisting of epithelioid cells is somewhat ill defined from surrounding tissue. Lymphocytes are seen surrounding the sarcoid granuloma, which is reminiscent of cellular immune reaction. Hematoxylin and eosin stain (under×40 magnification objective).

**Figure 4:**
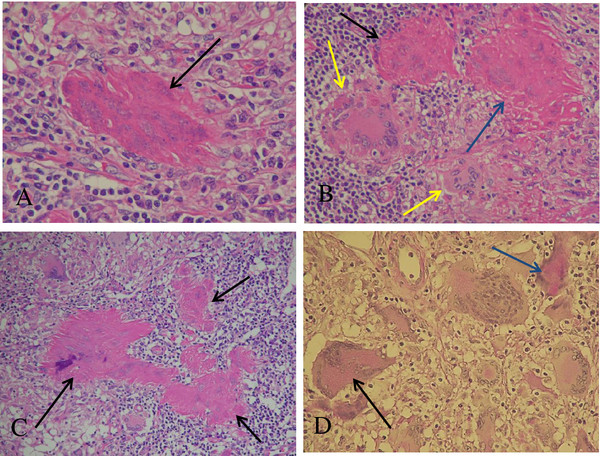
**Histopathology of the lymph node. (A)** Multiple fragmented condensed nuclei (apoptotic bodies) are seen in the eosinophilic degenerated giant cell (arrow) with fibrotic configuration. Hematoxylin and eosin stain (under×40 magnification objective). **(B)** Two eosinophilic degenerated giant cells (blue and black arrows) showing indistinct nuclear configurations, which are probably in the advanced stage of degeneration. One cell (blue arrow) shows fibrotic configuration. Yellow arrows indicate relatively intact giant cells. Hematoxylin and eosin stain (under×20 magnification objective). **(C)** Black arrows indicate three eosinophilic homogenous degenerated substances (hyaline-like degeneration) with fibrotic configuration. Nuclear remnants are barely visible. Morphological transition from degenerating/degenerated giant cells to hyaline-like degeneration is shown. Hematoxylin and eosin stain (under×20 magnification objective). **(D)** A degenerating giant cell (black arrow) and a degenerated giant cell (blue arrow) showing increased periodic acid–Schiff stainability. Periodic acid–Schiff stain (under×20 magnification objective).

**Figure 5:**
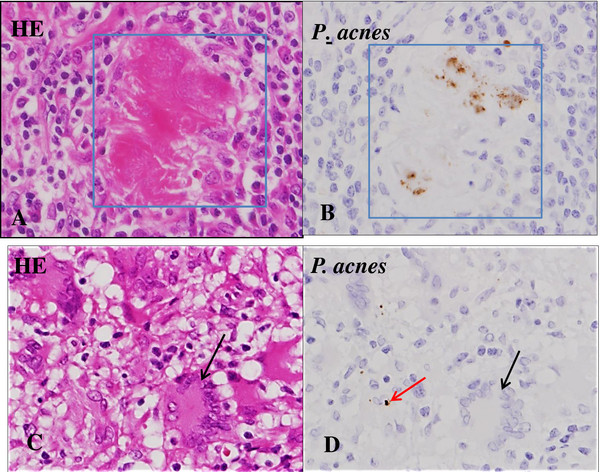
***Propionibacterium acnes *within sarcoid granulomas in the lymph node.** Hematoxylin and eosin staining (left) and immunostaining with PAB antibody specific to *Propionibacterium acnes* (counterstained with hematoxylin) (right) are shown pairwise. **(A, B)** Square indicates an eosinophilic homogenous degenerated substance (hyaline-like degeneration). Positive reaction products (brown dots) are located preferentially in the hyaline-like degeneration (under×40 magnification objective). **(C, D)** In the sarcoid granuloma, a small positive dot is seen in an epithelioid cell (red arrow). Some fine reaction products are sparsely scattered in the sarcoid granuloma. A black arrow indicates a Langhans giant cell (under×40 magnification objective). Abbreviations: HE, hematoxylin and eosin; *P*., *Propionibacterium*.

A right inguinal lymph node biopsy specimen, which was obtained 9 days after the initiation of CAM treatment, showed non-caseating epithelioid cell granulomas with abundant multinucleated giant cells (Langhans giant cells) in the background of epithelioid cells, macrophages, lymphocytes and plasma cells (Figure 3A). In the present case, the sarcoid granulomas were somewhat diminished in size and number, and ill defined from surrounding tissue (Figure 3B). In addition, some of the giant cells showed condensed heterochromatin (pyknosis; Figure 3A), multiple fragmented nuclei (apoptotic bodies; Figure 4A) and multiple nuclear remnants with indistinct nuclear configurations in the eosinophilic condensed cytoplasm (Figure 4B). These findings indicate that the giant cells were, although atypical, in the apoptotic process according to the features described by Wyllie *et al.*[7]. These degenerated giant cells appeared to have transformed into eosinophilic homogenous substances with fibrotic configuration (that is, hyaline-like degeneration; Figure 4C). The degenerating/degenerated giant cells showed slightly increased stainability with periodic acid–Schiff (Figure 4D). Neither acid-fast bacterium nor fungus was demonstrated by Ziehl–Neelsen and Grocott stains (not shown). Immunohistochemical staining for *Mycobacterium tuberculosis* showed a negative result (not shown). EBV-encoded small ribonucleic acid (RNA) was not detected in the sarcoid granulomas (not shown). To demonstrate the presence of *P. acnes* in the granulomas, the lymph node specimens were tested by immunohistochemistry using a specific monoclonal antibody against *P. acnes* lipoteichoic acid (PAB antibody). PAB-positive reaction products were observed preferentially in the degenerated homogenous substances. Small positive dots were scattered sparsely in the sarcoid granulomas (Figure 5).

**Figure 6:**
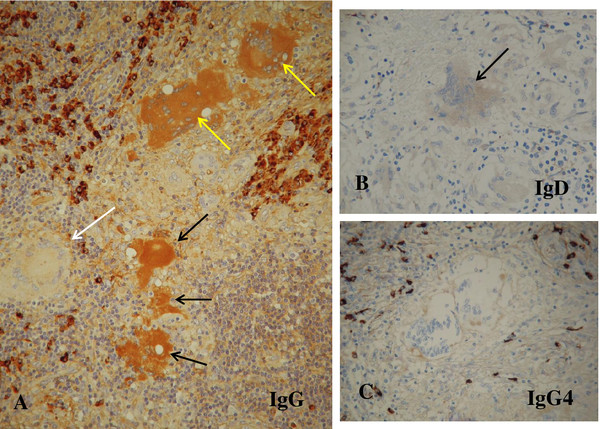
**Immunohistochemical staining of the lymph node for immunoglobulin G, immunoglobulin D and immunoglobulin G4. (A)** Degenerating giant cells (yellow arrows) and degenerated homogenous substance, possible hyaline-like degeneration (black arrows), are moderately positive for immunoglobulin G. By contrast, an intact giant cell (white arrow) is negative for immunoglobulin G. Infiltrated plasma cells are distinctly positive for immunoglobulin G (under×20 magnification objective). **(B)** The giant cell (arrow) is faintly positive for immunoglobulin D (under×20 magnification objective). **(C)** The giant cells are negative for immunoglobulin G4. The immunoglobulin G4-positive plasma cells are sporadic in frequency (under×20 magnification objective). Abbreviations: IgD, immunoglobulin D; IgG, immunoglobulin G; IgG4, immunoglobulin G4.

Since a considerable number of plasma cells were admixed with lymphocytes in the lymph node, immunohistochemical staining was carried out for immunoglobulin (Ig) G, IgA, IgM, IgD and IgG4 to dispel the possibility of IgG4-related disease (Figure 6). Infiltrated plasma cells were positive for IgG, IgA, IgM, IgD and IgG4 at different degrees. Positive reactions for IgG4 were only occasionally seen in the plasma cells, thus negating the possibility of IgG4-related disease. Some of the giant cells and degenerated homogenous substances were positively stained with anti-IgG antibody, and the reaction was more intense in degenerating/degenerated giant cells than in intact ones (Figure 6A). The giant cells were faintly stained with anti-IgD antibody (Figure 6B) but not with anti-IgA (not shown), anti-IgM (not shown) and anti-IgG4 (Figure 6C) antibodies.

**Figure 7:**
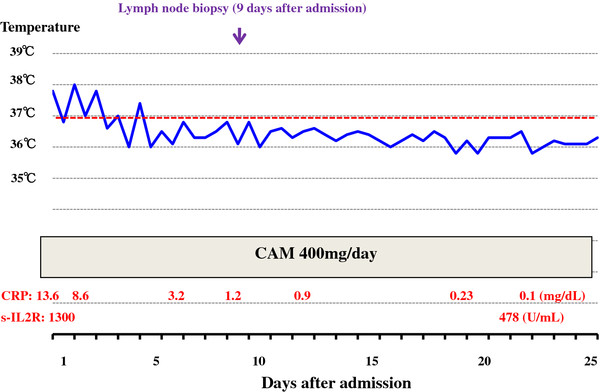
**Clinical course during admission.** After the initiation of clarithromycin treatment, the fever subsided within 1 week. The elevated levels of C-reactive protein returned to normal within 2 weeks and elevated soluble-interleukin-2 receptor levels were remarkably depressed within 3 weeks. Abbreviations: CAM, clarithromycin; CRP, C-reactive protein; s-IL2R, soluble-interleukin-2 receptor.

**Figure 8:**
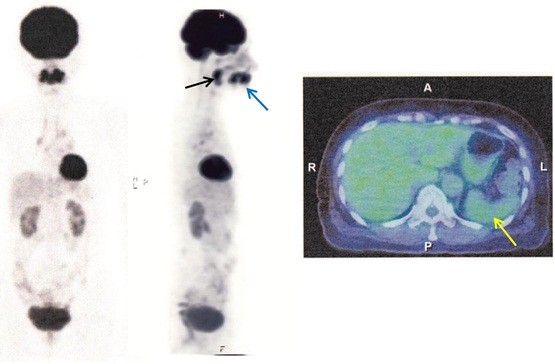
**Positron emission tomography and fused positron emission tomography-computed tomography images 2 months after admission.** Drastic resolution in axillary, mediastinal, iliac and inguinal lymph nodes is shown. The spleen (yellow arrow) shows no ^18^F-fluorodeoxyglucose uptake. However, submandibular dental roots (blue arrow) and peripharyngeal lymph nodes (black arrow) still show ^18^F-fluorodeoxyglucose uptake.

In this case, an oral administration of CAM (200mg at 12-hour intervals) coupled with acetaminophen (400mg at 12-hour intervals) was empirically initiated on the day of admission. After the initiation of this treatment, her fever rapidly subsided coupled with the disappearance of joint pains and toxic eruptions within 1 week. The elevated levels of CRP returned to normal within 2 weeks and elevated s-IL2R levels were remarkably decreased within 3 weeks (Figure 7). She was discharged 25 days after admission and followed as an out-patient. Administration of acetaminophen was suspended at the time of discharge. The levels of s-IL2R returned to normal within 2 months. The follow-up PET-CT scan 2 months after admission showed minimal FDG uptake in her bilateral hilar lymph nodes and distinct uptake at submandibular dental roots and in peripharyngeal lymph nodes. The FDG uptake in her spleen also disappeared (Figure 8). CAM treatment was discontinued 3.5 months after its initiation. No unfavorable adverse effects were observed with CAM. During the course of illness, the patient was found to have urinary bladder cancer, which was successfully removed by endoscopy in the urologic department of another hospital. Her levels of s-IL2R remained slightly elevated (less than 750IU/mL) after cessation of CAM treatment without any symptoms. She stopped visiting our hospital 1.5 years after initial presentation because of traffic inconvenience to visit the hospital. Recently, she visited our hospital to undergo medical check-ups and was told that she was in good condition.

## Discussion

Although the exact cause of sarcoidosis is still unknown, the current working hypothesis suggests that it is caused by alteration of the immune response after exposure to environmental, occupational or infectious agents in genetically susceptible individuals. Among the infectious agents, mycobacterial and propionibacterial organisms are most commonly implicated as potential etiologic agents [1-4,8-10]. In particular, *P. acnes* is the only microorganism that has been isolated from sarcoid lesions [4]. Many fragments of *P. acnes* nucleic acids have been detected in sarcoid lymph nodes by quantitative polymerase chain reaction [11-13] and *in situ* hybridization [13]. All available evidence has suggested the concept that the disease results from an exaggerated Th1 immune response to specific antigens [1,2,8,14]. Moller *et al.* suggested that IL-12 induction and amplification of Th1 responses probably play an important role in the granuloma development in sarcoidosis through the effect of interferon-γ (IFN-γ) on macrophage activation [15]. The fact that the sarcoid granulomas can be experimentally induced in mice by administration of a recombinant trigger-factor protein of *P. acnes* lends supportive evidence for hypersensitivity to *P. acnes*[16].

In the present case, *P. acnes* was detected immunohistochemically using PAB antibody. Positive reaction products were found preferentially in eosinophilic degenerated homogenous substances. The presence of *P. acnes* in the lymph node seems to be the supportive evidence for diagnosing sarcoidosis. Furthermore, its presence in the lymph node indicates that the sarcoidosis was caused by *P. acnes* infection. The pathology of the lymph node in this case was key to understanding the degenerative process of Langhans giant cells. Some of the degenerating/degenerated giant cells showed apoptotic features. In addition, morphological transition from degenerated giant cells to hyaline-like substances was also observed. Thus, many giant cells were seen to undergo apoptosis due to the CAM treatment. Taking into account the apoptosis in the giant cells, it is possible that epithelioid cells might also have been involved in apoptosis. However, it was difficult to identify apoptotic features in individual epithelioid cells in the granulomas at the light microscopic level. The presence of IgG and IgD in the giant cells might be related to the apoptotic process because IgG was stained more intensely in the degenerating/degenerated giant cells as well as in the hyaline-like substances than in the intact giant cells. The presence of IgG and IgD in the giant cells might be due to incorporation of Igs by endocytosis or increased permeability of the cell membrane. In this connection, the incorporation of M-protein (IgG), possibly by endocytosis, has been demonstrated by immunoelectron microscopy in various bone marrow cells such as erythroblasts, monocytes, reticulum cells, megakaryocytes, and stromal cells in a case of IgG-κ type multiple myeloma [17]. Thus, it is not surprising that the IgG and IgD were detected immunohistochemically in the giant cells. Non-specific reaction for IgG and IgD seems improbable because no positivity for IgG4, IgA, and IgM was demonstrated in the giant cells by the same immunohistochemical procedure. The exact mechanism and the significance of incorporated Igs remain to be elucidated, but this may reflect the degree of degeneration of the giant cells.

In the present case, typical clinical features for sarcoidosis such as pulmonary, ocular or cutaneous lesions were not observed. Moreover, her levels of serum angiotensin-converting enzyme and lysozyme were not elevated. However, the PET-CT scan clearly showed axillary, mediastinal, hilar, iliac and inguinal lymph node swellings with massive splenic involvement. The diagnosis of sarcoidosis was made histopathologically based on the presence of non-caseating epithelioid granulomas with abundant Langhans giant cells in her lymph node. Although histologic evidence is mandatory for a definitive diagnosis of sarcoidosis, the histologic findings are not sufficiently specific to make the diagnosis, since non-caseating granulomas are found in a number of infectious diseases. In the present case, such a possibility was ruled out by negative serologic tests for *Brucella*, *Legionella*, *Coxiella burnetii, Mycoplasma* and *Toxoplasma* species. Serologic tests for EBV and *Chlamydia pneumoniae* showed prior infection patterns. In addition, EBV-encoded small RNA was negative in the sarcoid granulomas. Legionella infection was unlikely because the patient had no pneumonia at presentation. Leishmaniasis is also unlikely because this disease is non-endemic in Japan and parasitized histiocytes were not observed in the biopsied lymph node. Although *P. acnes* seems to be a potential etiologic agent, other viral, bacterial and environmental agents and genetic susceptibility might be also involved in the development of sarcoidosis in a complex manner.

In general, sarcoidosis can either remit spontaneously or become chronic, with exacerbation and remission. Two-thirds of the patients with sarcoidosis generally have a remission within a decade after diagnosis with few or no consequences. Remission occurs in more than half of the patients within 3 years [2]. In most cases, patients with sarcoidosis are not disabled by the illness, so the decision and selection of its treatment should be provided with discretion. A variety of drugs including corticosteroids, methotrexate (MTX), azathioprine, thalidomide, cyclophosphamide, cyclosporine, hydroxychloroquine, chloroquine, nonsteroidal anti-inflammatory drugs and anti-tumor necrosis factor (TNF)-α blockers have been tried [2]. Bachelez *et al.*[18] reported that in 12 patients with cutaneous sarcoidosis treated with minocycline, a clinical response was observed in 10 patients, which consisted of complete responses in eight patients and partial response in two patients. The use of minocycline and its analogues have also been proposed because of their antimicrobial and immunomodulatory effects [18]. Recently, it was reported that minocycline treatment for sarcoidosis reduced the circulating levels of IL-12p40 and interferon (IFN)-inducible protein-10, thus emphasizing the immunomodulating rather than antimicrobial effects of the drug [19]. In the present case, we started CAM treatment without a definite diagnosis of sarcoidosis at the time of admission. The diagnosis of sarcoidosis was only confirmed pathologically 2 weeks after the initiation of CAM treatment. Soon after the initiation of this treatment, CAM was found to be effective; the fever rapidly subsided with depressed levels of CRP and s-IL2R. Baba *et al.*[20] reported a case of sarcoidosis with multiple endobronchial mass lesions which disappeared after treatment with CAM coupled with minocycline. However, there has been no report documenting a drastic effect for sarcoidosis after a single CAM administration. Although acetaminophen might have played a role in depressing the fever to a certain extent, the drug itself has no immunomodulatory or immunosuppressive effect. Thus, CAM was thought to have played a major role in improving sarcoidosis.

Recently, we reported a case of MTX-related EBV-associated Hodgkin-like lymphoma, in which complete remission was achieved after withdrawal of MTX coupled with CAM administration [21]. We emphasized that CAM has important pharmacological effects such as immunosuppression or immunomodulation in addition to an antimicrobial role. It is postulated that CAM can influence the cytokine networks and enhance the activities of natural killer cells and cytotoxic T-cells [22]. Many investigators have reported immunosuppressive or immunomodulatory effects of CAM in patients with cancers [23,24] or tumor-bearing animals [22]. In brief, CAM can decrease the production of IL-1 [5,21,25], IL-2 [6,26,27], IL-5 [26], IL-6 [5,6,21,24,26,27], IL-8 [6,28], TNF-α [5,21,24,26,29], transforming growth factor-β [29] and matrix metalloproteinase 9 [29], and increase the production of IL-4 [22], IL-12 [23] and IFN-γ [22,23]. In the present case, the level of cytokines was not investigated. However, the pathology of the biopsied lymph node was very suggestive to understand the mode of action by CAM; the sarcoid granulomas clearly showed apoptotic degeneration in Langhans giant cells. In this connection, CAM has been reported to induce apoptosis of activated human lymphocytes [30]. Here, attention should be paid to the fact that the lymph node biopsy was carried out 9 days after the initiation of CAM treatment. This means that CAM had already exerted its pharmacological effect on the sarcoid granulomas at the time of biopsy. In other words, pathological findings of the lymph node represented the healing process by CAM treatment.

In the present case, the PET-CT images consistently showed distinct FDG uptake at the submandibular dental roots, suggesting the presence of chronic periodontitis. Concomitant FDG uptake in peripharyngeal lymph nodes seemed to be related with chronic periodontitis. *P. acnes* is known to be indigenous to the skin, it is also considered to be important in the etiology of periodontal diseases. In this case, the possibility that the chronic periodontitis might have been a focus of *P. acnes* infection cannot be ruled out.

## Conclusions

The present case provided helpful clues in the treatment of *P. acnes*-associated sarcoidosis. CAM was found to be effective for treating sarcoidosis, and seems to have important pharmacological effects such as immunosuppression, immunomodulation and induction of apoptosis in addition to its antimicrobial role. The pathological analysis of the lymph node, which was obtained during the CAM treatment, demonstrated an apoptosis-inducing effect of CAM on the sarcoid granulomas. This effect might have resulted in satisfactory improvement in this case.

## Consent

Written informed consent was obtained from the patient for publication of this case report and accompanying images. A copy of the written consent is available for review by the Editor-in-Chief of this journal.

## Abbreviations

BHL: Bilateral hilar lymphadenopathy; CAM: Clarithromycin; CRP: C-reactive protein; CT: Computed tomography; EBV: Epstein–Barr virus; FDG: ^18^F-fluorodeoxyglucose; IFN-γ: Interferon-γ; Ig: Immunoglobulin; IL: Interleukin; MTX: Methotrexate; PET: Positron emission tomography; RNA: Ribonucleic acid; s-IL2R: Soluble-interleukin-2 receptors; TNF: Tumor necrosis factor.

## Competing interests

The authors declare that they have no competing interests. All authors have no sources of funding from CAM-related pharmaceutical companies.

## Authors’ contributions

NT carried out the clinical treatment, follow-up and collecting data, and was also a major contributor in writing the manuscript. MN carried out the lymph node biopsy. MK and YE performed the histochemistry and immunohistochemistry and are the major contributors for the pathological diagnosis. All authors read and approved the final manuscript.
